# Supplementary material for: FLASHIda enables intelligent data acquisition for top–down proteomics to boost proteoform identification counts
Source: Nat Commun. 2022 Jul 29;13:4407. doi: 10.1038/s41467-022-31922-z (PMC9338294; doi:10.1038/s41467-022-31922-z)
Supplement: Supplementary file 1 — Supplementary information [file 41467_2022_31922_MOESM1_ESM.pdf]

Supplementary information for “FLASHIda enables intelligent data acquisition for top-down proteomics to boost proteoform identification counts”

Kyowon Jeong[1,2,*], Maša Babović[3], Vladimir Gorshkov[3], Jihyung Kim[1,2], Ole N. Jensen[3], and Oliver Kohlbacher[1,2,4,*]

kyowon.jeong@uni-tuebingen.de
oliver.kohlbacher@uni-tuebingen.de

[1] Applied Bioinformatics, Department for Computer Science, University of Tübingen, Sand 14, 72076 Tübingen, Germany

[2] Institute for Bioinformatics and Medical Informatics, University of Tübingen, Sand 14, 72076 Tübingen, Germany

[3] Department of Biochemistry & Molecular Biology and VILLUM Center for Bioanalytical Sciences, University of Southern Denmark, Campusvej 55, DK-5230 Odense M, Denmark

[4] Translational Bioinformatics, University Hospital Tübingen, Hoppe-Seyler-Str. 9, 72076 Tübingen, Germany

[*]Corresponding authors

Supplementary Figure 1.

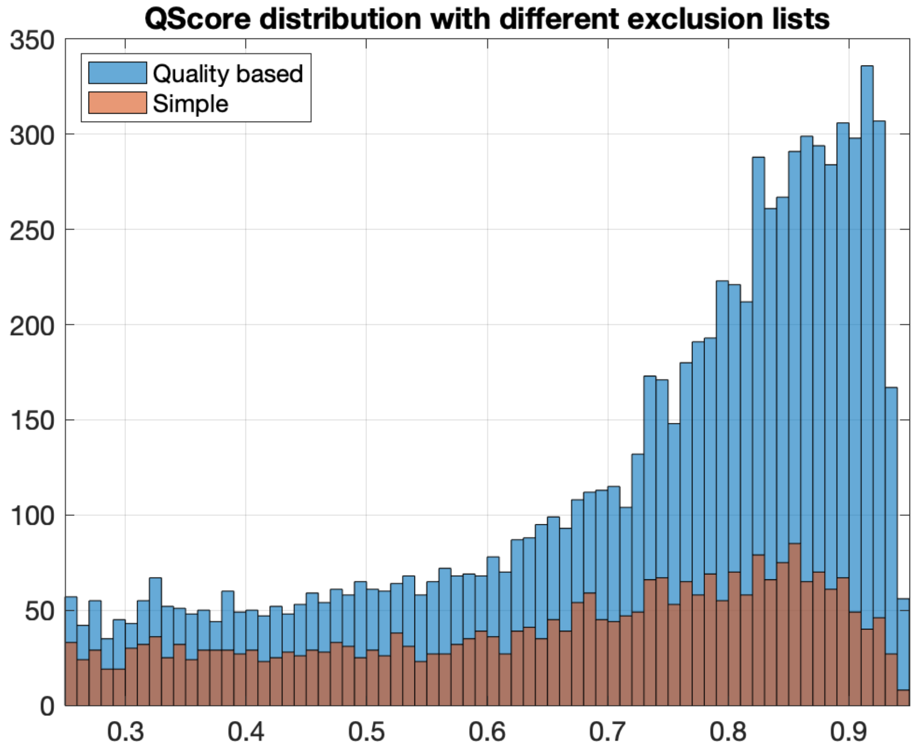

Supplementary Figure 1. QScore distribution comparison between quality-based exclusion list and simple mass exclusion list in FI90 dataset. The quality-based exclusion resulted in 8,416 acquired precursors whereas the simple mass exclusion resulted in only 2,860 precursors. Moreover, significantly more spectra have higher QScores with the quality-based exclusion list than with the simple mass exclusion list. The number of identified spectra can be estimated simply by adding up all QScores of spectra, because QScore is the estimated identification probability. The estimated number of identified spectra for quality-based exclusion is 6,096 while it is 1,884 for simple mass exclusion list. Note that the actual identified spectra for FI90 is 5,805. Source data are provided as a Source Data file.

Supplementary Figure 2.

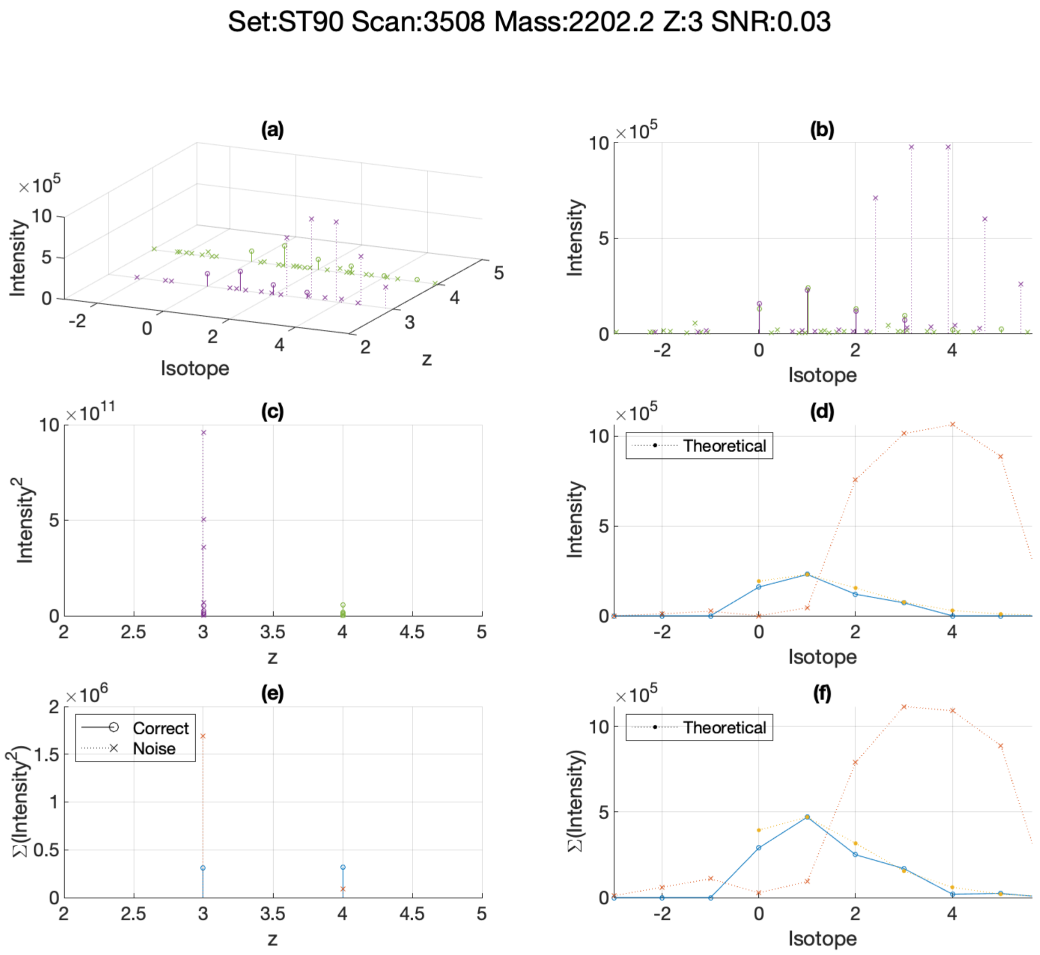

Supplementary Figure 2. Selected precursors of low SNR (<1.0) from ST90 dataset: an example of coelution. The MS1 signals are shown for a representative precursor of low SNR. The figure title shows the dataset (Set:), scan number (Scan:), monoisotopic mass (Mass:), precursor charge (Z:), and precursor SNR (SNR:). **(a)** all peaks within isotopomer envelopes of distinct charges for the precursor monoisotopic mass. x-axis (Isotope) shows the isotope index, y-axis (z) shows the charge state, and z-axis (Intensity) the peak intensity. The circled peaks (or correct peaks) are the peaks corresponding to the isotopes of the mass, and crossed peaks (noise peaks) are the remaining peaks. **(b)** is the same as **(a)**, but viewed perpendicular to the x-y (isotope-intensity) plane, showing all peaks along isotope indices. **(c)** shows the squared intensity, or power, of individual peaks along charges. **(d)** shows the peak intensity along isotope indices only for the precursor charge (in this figure, z=3). All peaks are binned into the closest isotope indices. The blue (red) line shows the observed envelope from the correct (noise) peaks, and the yellow line is the theoretical envelope. **(e)** is drawn from **(c)** by aggregating the power of correct peaks (blue) and that of noise peaks (red) along charges. **(f)** is analog of **(d)** with the aggregated peak intensity for all charge states. The signal above is an example of coelution. **(a)** and **(b)** show explicit coelution (peaks for z = 3). In **(c)** and **(e)**, it is shown that noise power exceeds signal power for the precursor charge (z = 3). **(d)** and **(f)** show the presence of two distinct coeluted envelopes.

Supplementary Figure 3.

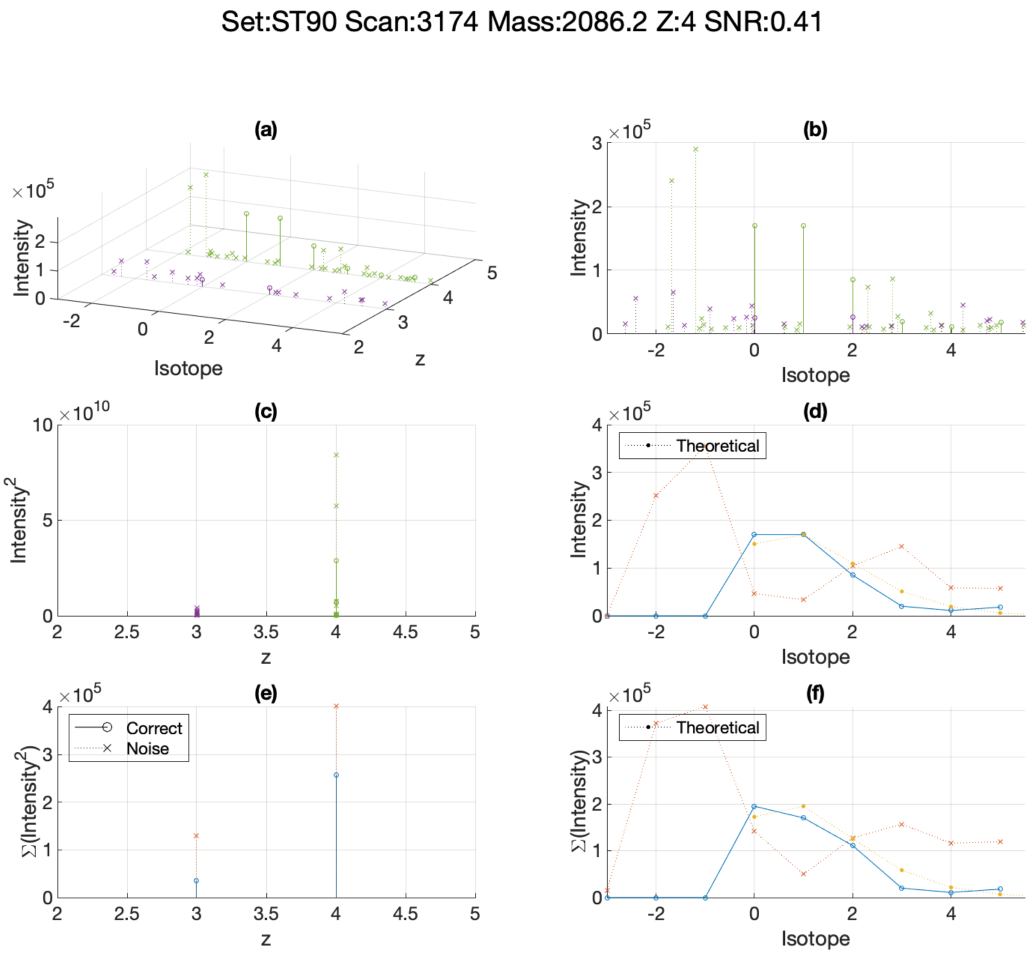

Supplementary Figure 3. Analog of Supplementary Fig. 2 (a-f) for selected precursors of low SNR (<1.0) from ST90 dataset: another example of coelution. Two envelopes of different charges are present within the range of the precursor envelope (z = 4).

Supplementary Figure 4.

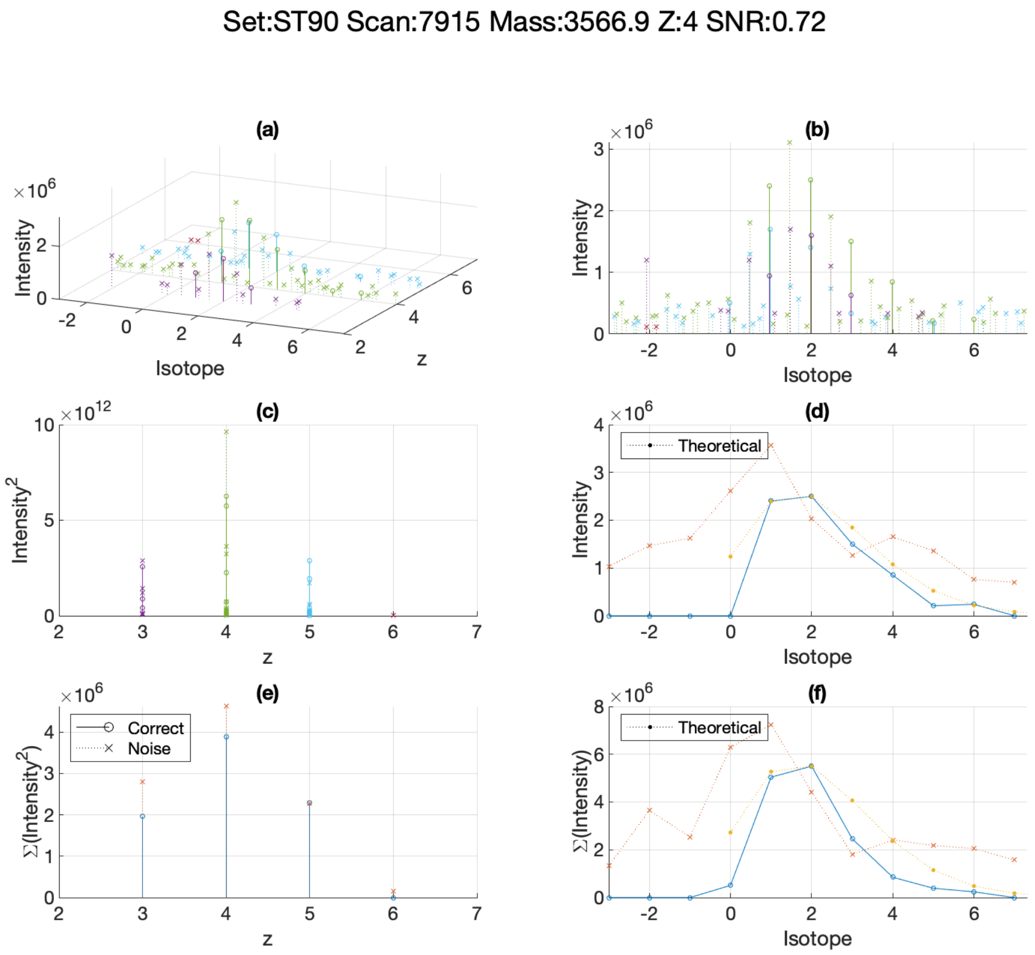

Supplementary Figure 4. Analog of Supplementary Fig. 2 (a-f) for selected precursors of low SNR (<1.0) from ST90 dataset: an example of harmonic artifact. The determined charge for the precursor is four, but from **(b)**, it is obvious that the correct precursor charge should be eight. The wrongful assignment of the precursor charge results in low SNR, which enables the removal of such artifacts. Signals of all low SNR precursors from ST90 dataset are deposited in MassIVE under accession number MSV000087484 [https://massive.ucsd.edu/ProteoSAFe/dataset.jsp?task=c2f199a1a5874350b48aea2fcb16c505] or under the digital object identifier [https://doi.org/10.25345/C5FJ9G].

Supplementary Figure 5.

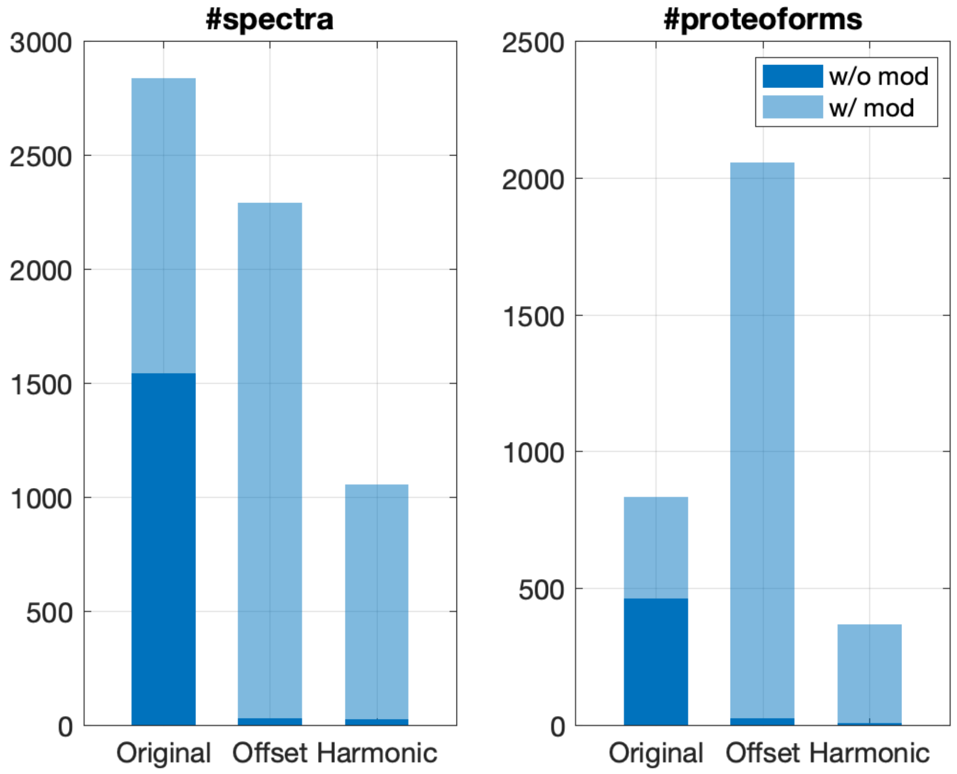

Supplementary Figure 5. Numbers of identified spectra and proteoform IDs in ST90 dataset with intentionally introduced precursor mass errors. The numbers of identified spectra and unique proteoform IDs from ST90 dataset are shown for three different cases: MS2 spectra of original precursor masses reported by FLASHDeconv (Original in x-axis label), original masses plus non-zero random offsets from 1 to 10 or -1 to -10 (Offset), and original masses divided by two simulating low harmonic artifacts (Harmonic). The bright blue portion indicates the portion of identification with an unknown modification. The identification was done with TopPIC at 1% spectrum and proteoform level FDR estimated by target-decoy database. While the original precursor masses resulted in the most identified spectra, the ones with offsets resulted in the largest number of proteoform IDs. This is because proteoform IDs with incorrect precursors usually are considered as "unique novel" proteoform IDs. In the harmonic simulation dataset as well, more than thousand spectra were identified (out of total 6,476), and hundreds of proteoform IDs were identified, even if most of them represent obvious false positives. Also, except for the original dataset, most identifications had unknown mass shifts (as expected from the spectra of incorrect precursor masses). This result clearly shows that incorrect precursor masses not only are hard to reduce via target-decoy approach but also may inflate the number of unique proteoform IDs. The identification results are found in Supplementary Data 1. Source data are provided as a Source Data file.

Supplementary Figure 6.

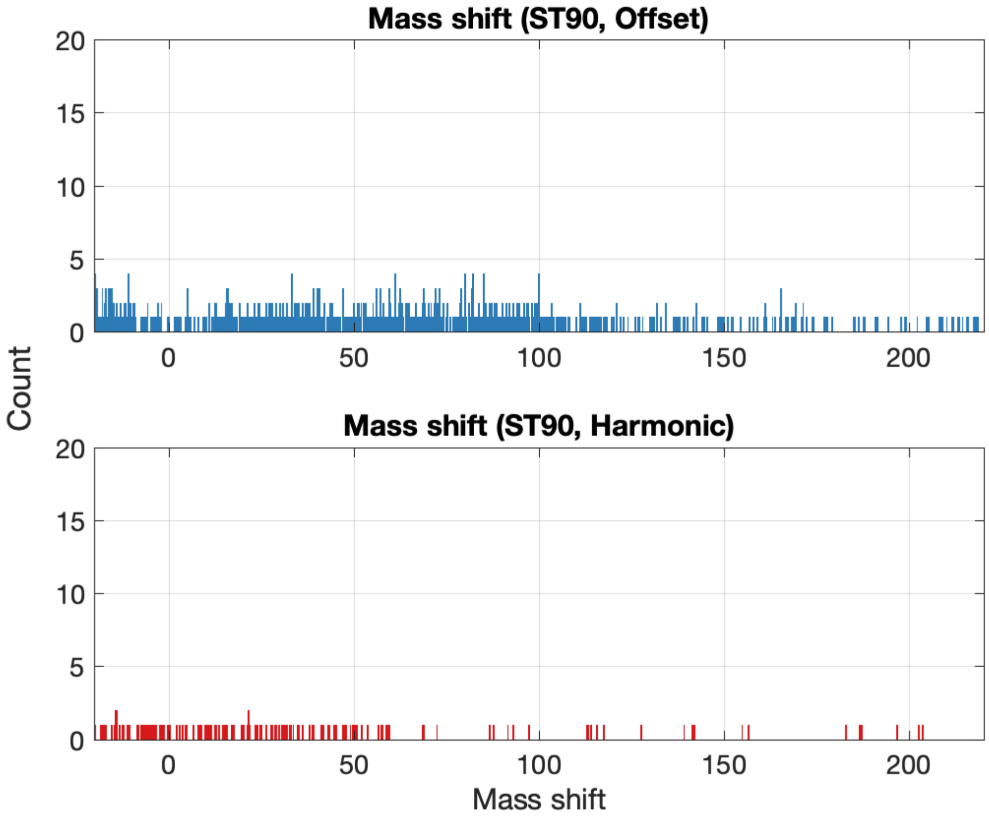

Supplementary Figure 6. Analog of Fig. 3e for proteoform IDs of incorrect precursors. The histograms of the mass shifts in unique proteoforms from the datasets in Supplementary Data 1 are shown. Top panel is for the precursors with random offsets (Offset in Supplementary Fig. 5) and bottom for the precursors divided by two (Harmonic in Supplementary Fig. 5). When compared to Fig. 3e, no high frequency mass shifts are observed, as expected from false positive hits. Source data are provided as a Source Data file.

Supplementary Figure 7.

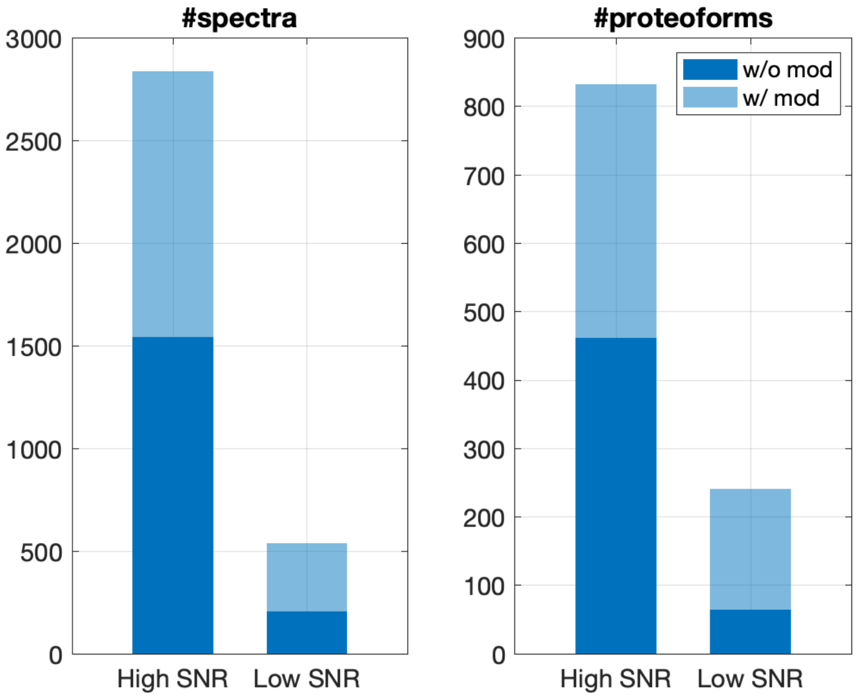

Supplementary Figure 7. Analog of Supplementary Fig. 2 for low precursor SNR identifications. ST90 dataset was identified without precursor SNR filtration, and the identified spectra and proteoform IDs were divided into two groups: from high-SNR precursors (precursor SNR>1; High SNR in x-axis) and from low-SNR precursors (precursor SNR<1; Low SNR). The left panel shows the number of identified spectra from each group and the right that of proteoform IDs. For the high SNR group, about 50% of spectral and proteoform IDs have no modification. But for the low SNR group, only 38% spectra and 27% proteoform IDs have no modification. Source data are provided as a Source Data file.

Supplementary Figure 8.

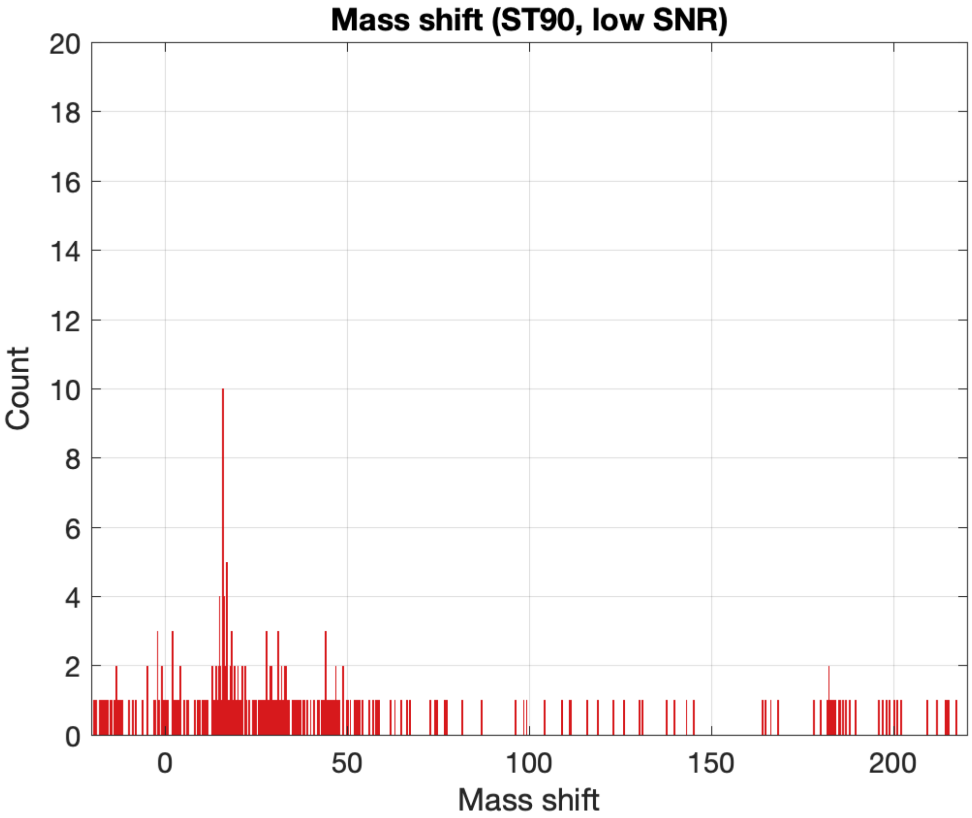

Supplementary Figure 8. Analog of Fig. 3e for low precursor SNR proteoform IDs. The histogram of the mass shifts of the proteoform IDs in the low SNR group in Supplementary Fig. 7 is shown. Except for Oxidation, no high frequency mass shifts are observed when compared to the high SNR group in Fig. 3e bottom panel. Together with Supplementary Fig. 7, this result suggests that a large portion of proteoform IDs of low SNR precursors represent false positives. Source data are provided as a Source Data file.

Supplementary Figure 9.

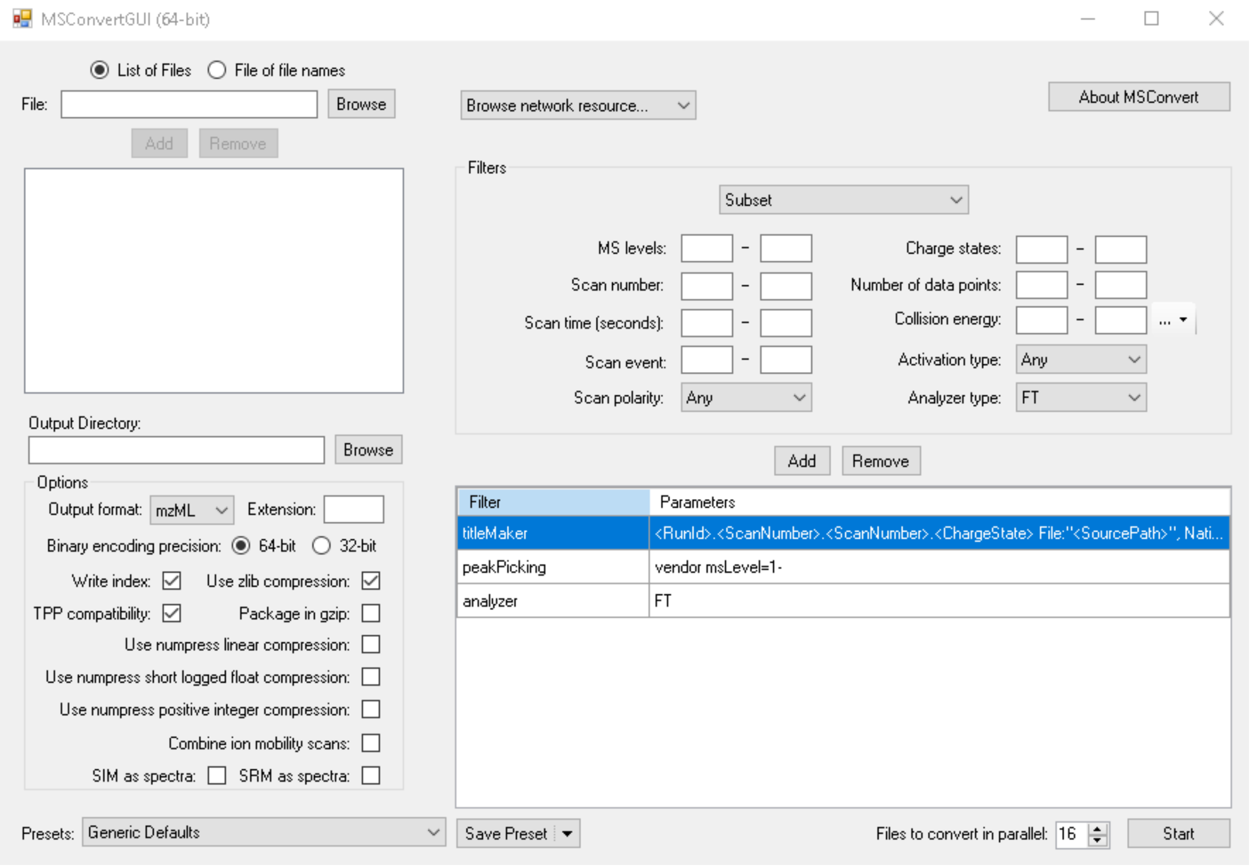

Supplementary Figure 9. **Screen capture of msconvert GUI displaying runtime parameters.** Thermo raw files containing profile mode spectra are converted into mzML format files by msconvert. All converted files are deposited in MassIVE under accession number MSV000087484 [https://massive.ucsd.edu/ProteoSAFe/dataset.jsp?task=c2f199a1a5874350b48aea2fcb16c505] or under the digital object identifier [https://doi.org/10.25345/C5FJ9G].

Supplementary Figure 10.

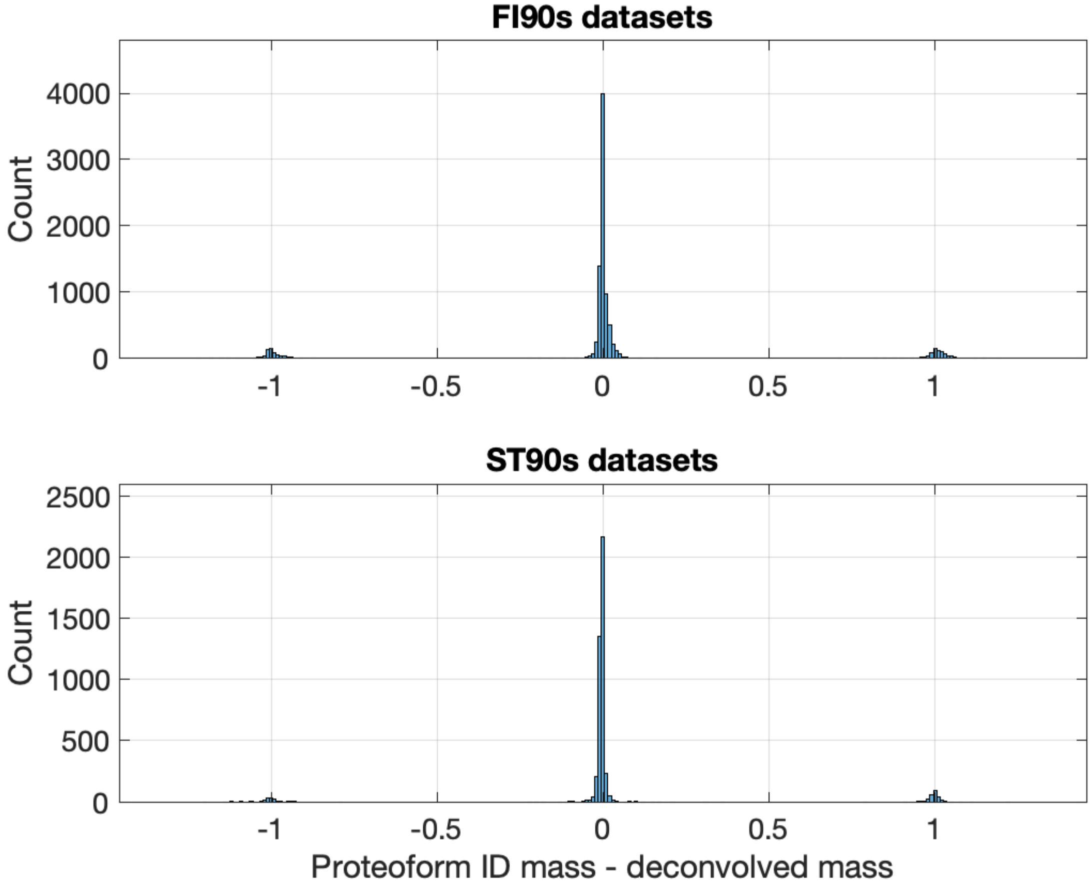

Supplementary Figure 10. The histogram of mass difference between precursor and proteoform ID for FI90s and ST90s datasets. To see if the increased unique precursor mass count is due to common one Da mass error arising from inaccurate deisotoping in precursor mass deconvolution, we examined the mass difference between deconvolved precursor mass and proteoform mass determined by identification in FT90s (upper) and ST90s (lower panel) datasets. The x-axis shows proteoform ID mass values (Adjusted precursor mass column) subtracted by deconvolved precursor mass values (Precursor mass column in TopPIC output tsv files). Standard DDA and FLASHIda generated comparable ratios of one Da mass error (7.2% and 9.9% in ST90s and FI90s datasets, respectively), where one Da mass error is defined to occur when the absolute value of the mass difference in x-axis is within 10 ppm mass tolerance. Source data are provided as a Source Data file.

Supplementary Figure 11.

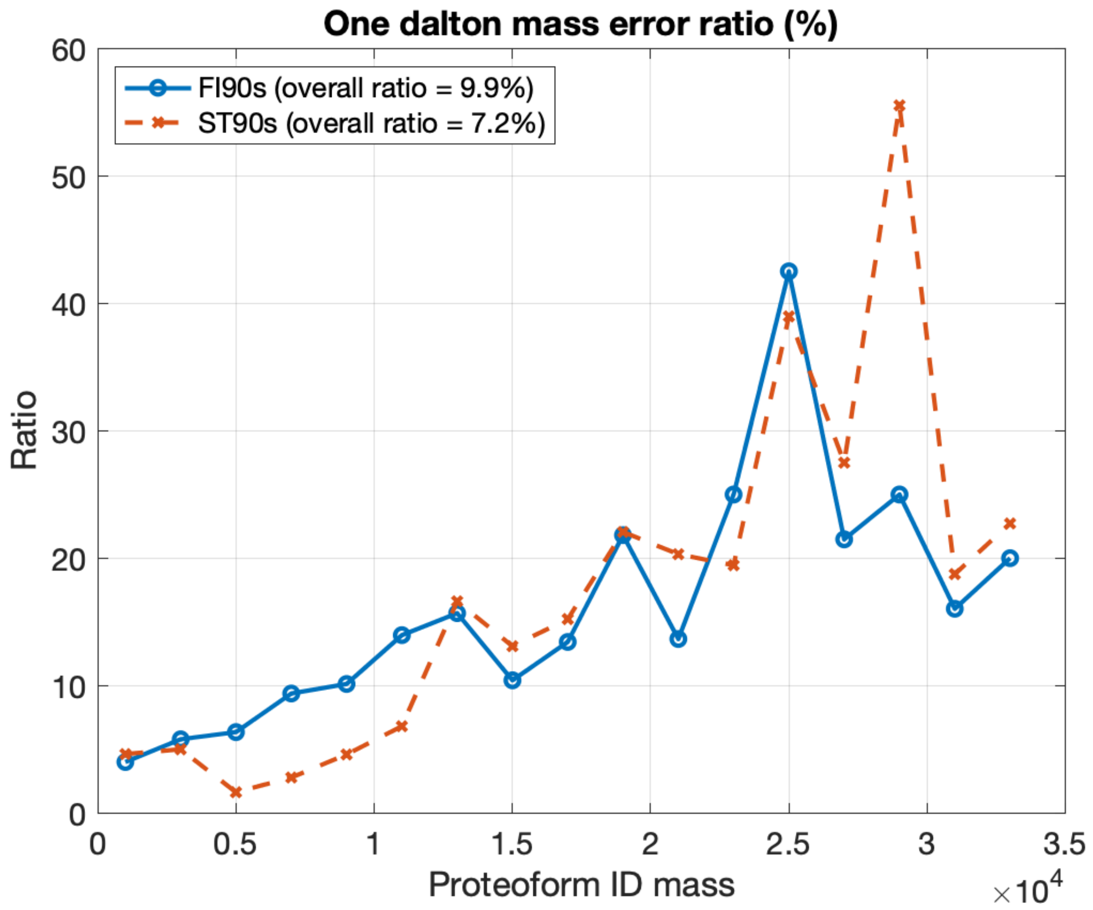

Supplementary Figure 11. One Da mass error rates along proteoform masses for FI90s and ST90s datasets. The one Da mass error (defined in Supplementary Fig. 10) ratio is drawn for different ranges of proteoform ID masses. The ratio (in %) is defined by (#proteoform IDs with one Da mass errors divided by #all proteoform IDs) per mass range. FLASHIda showed less error ratio than standard DDA for heavy proteoform IDs (>25 kDa). Source data are provided as a Source Data file.

Supplementary Figure 12.

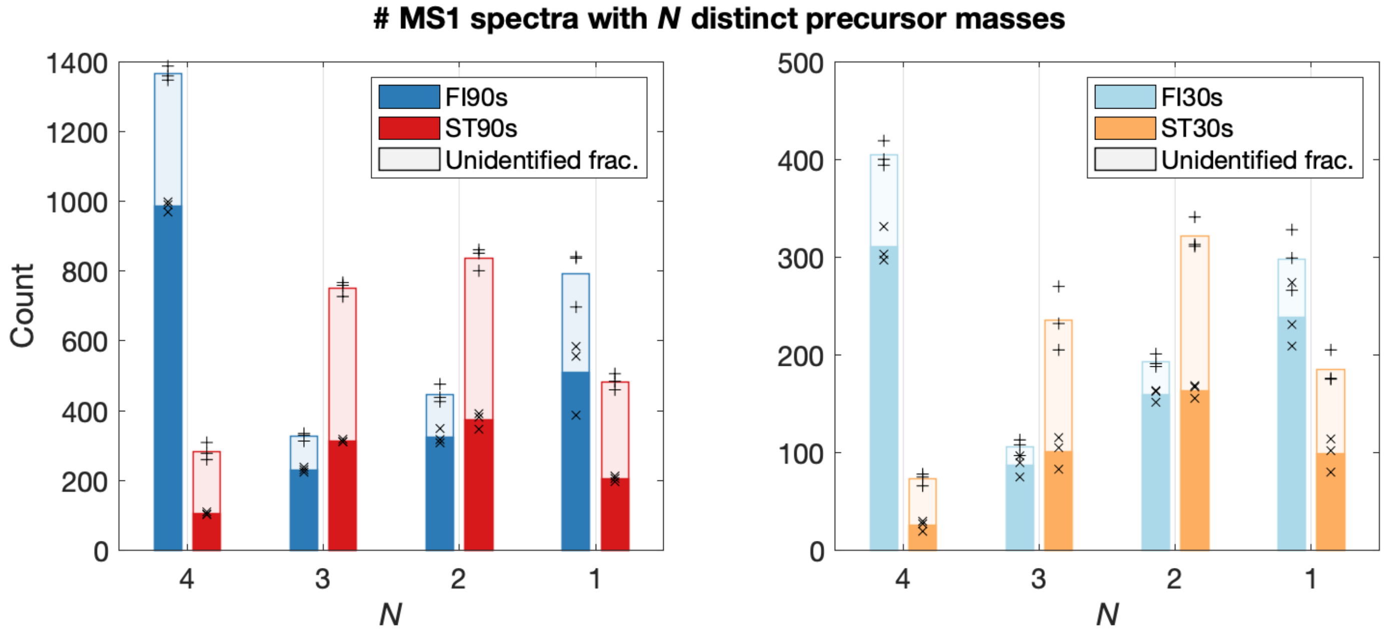

Supplementary Figure 12. The number of distinct precursor masses triggered in MS1 spectra. To more clearly observe if Top-4 QScore selection works as intended, we plot how many distinct masses were triggered per MS1 spectrum and how many out of them were identified. n=3 technical replicates were examined over the four independent single LC-MS/MS runs (FI90s, ST90s, FI30s, and ST30s datasets) from the *E. coli* lysate sample.

The (+) markers show the numbers of all MS1 spectra, and the (x) markers show the numbers of identified MS1 spectra from replicates. The bars show their average values. FLASHIda selected four distinct masses almost five times more often than the standard acquisition even though the numbers of total MS2 spectra from FLASHIda were about 20% less than from the standard for all datasets (seen in Fig. 2a, right panel). The portion of the identified spectra was also higher for FLASHIda than for the standard runs in all cases, regardless of the number of distinct masses. Source data are provided as a Source Data file.

Supplementary Figure 13.

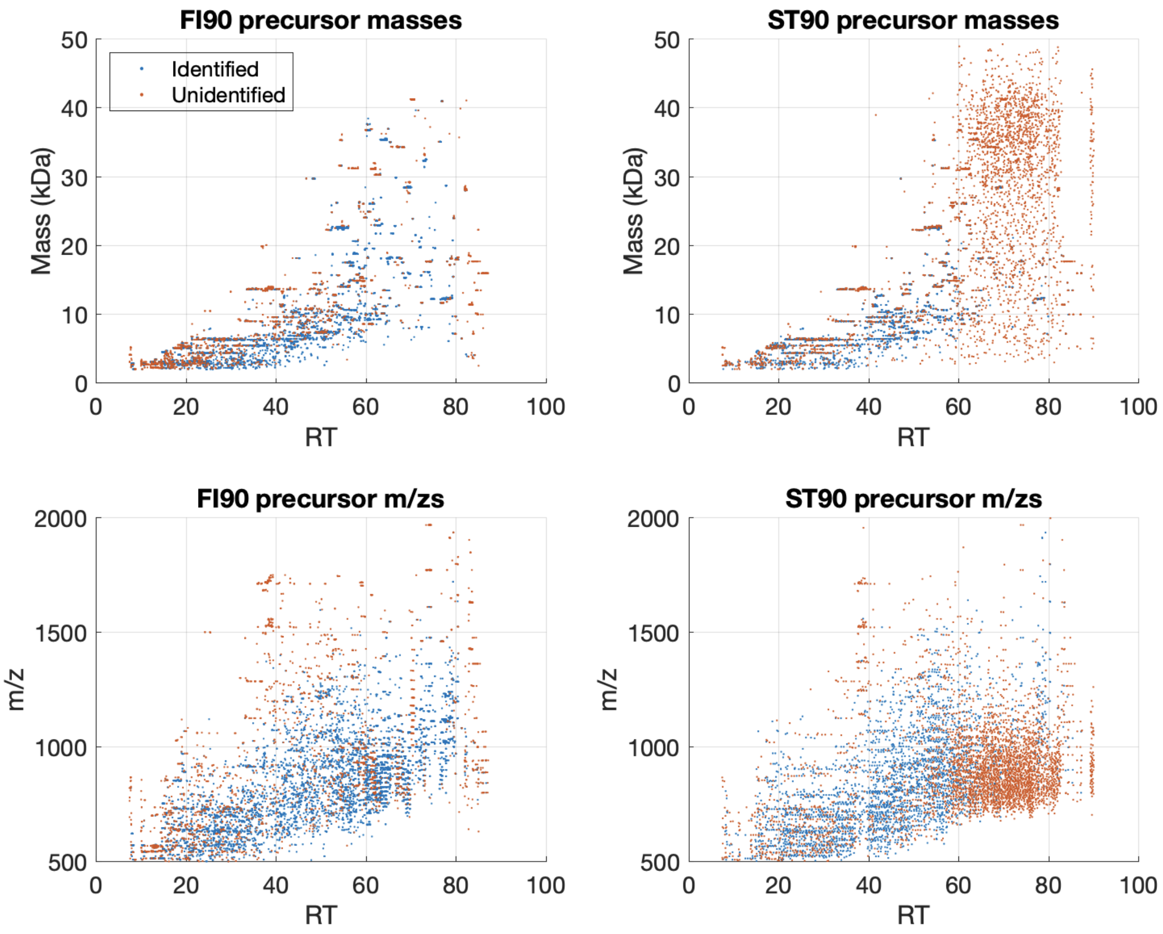

Supplementary Figure 13. Scatter plots of identified and unidentified precursors on RT-mass and RT-m/z planes for FI90 and ST90 datasets. The blue dots are the identified spectra and red unidentified. For both datasets, distributions on the RT-mass plane (upper panels) are far more sparse than on the RT-m/z plane (lower). In the 60-80 minute RT range, FLASHIda (left panels) did not acquire many MS2 spectra due to lack of high quality precursors. But the standard acquisition (right) generated many MS2 spectra in the same RT range, most of which resulted in unidentified spectra. While this shows the specific precursor selection of FLASHIda, it also shows that the identification of high mass proteoforms should be improved, for example, using other dissociations like ETD for high mass precursors.

Supplementary Figure 14.

(a)

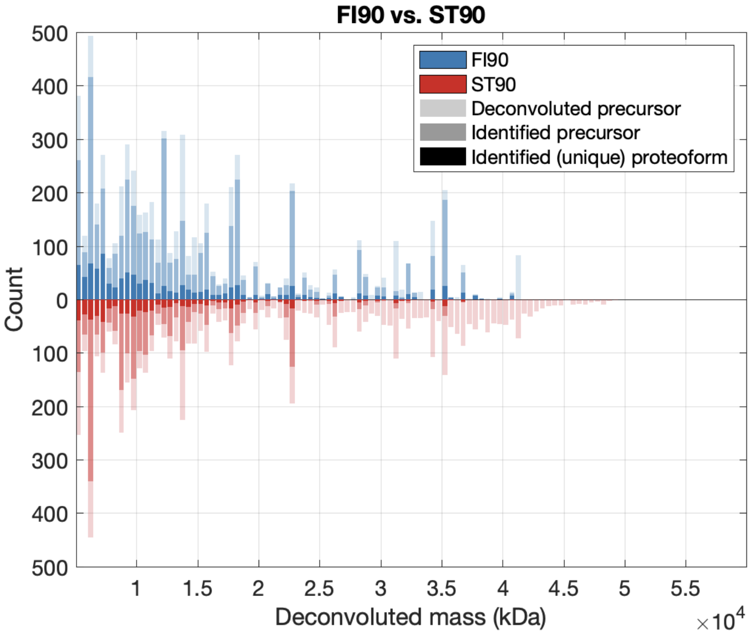

(b)

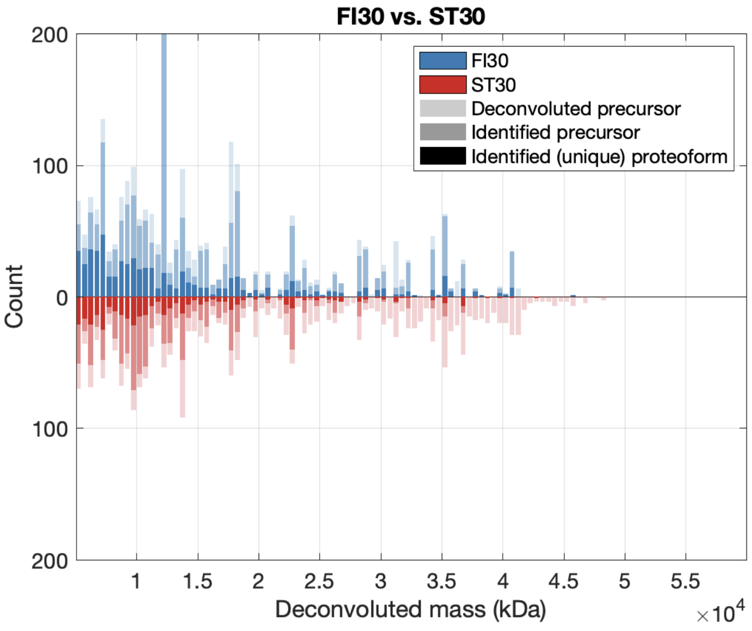

Supplementary Figure 14. Mass distribution and identification rate for FI90 vs. ST90 (a) and FI30 vs. ST30 (b). The blur bars are for FI and red for ST datasets. Each bar shows the number of deconvoluted spectra (high bright), identified spectra (medium bright), and unique proteoform IDs (dark). These plots show that even though the standard acquisition triggers more large masses (> 20 kDa) than FLASHIda does, FLASHIda identifies more high masses. Source data are provided as a Source Data file.

Supplementary Figure 15.

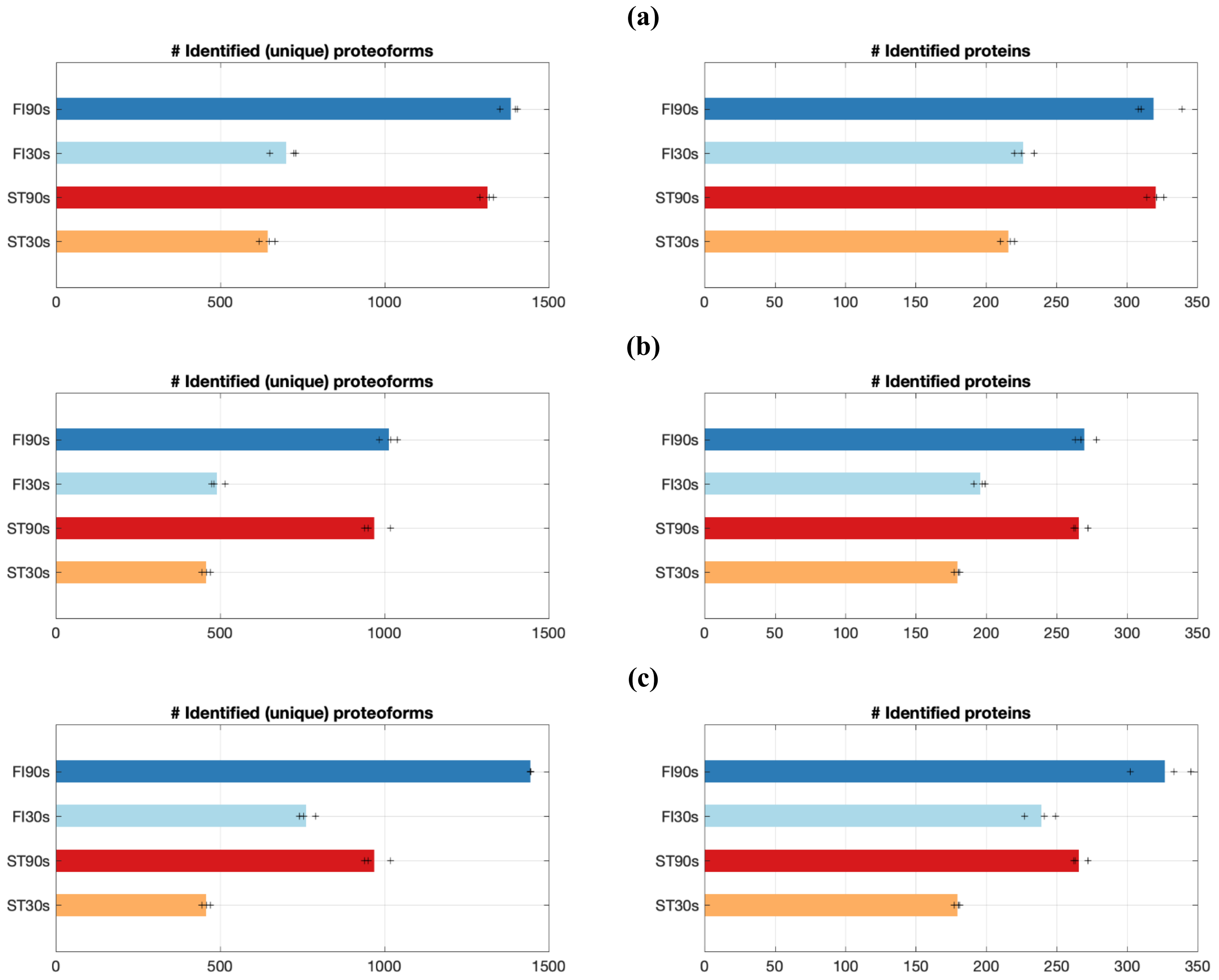

Supplementary Figure 15. Identification of FI and ST datasets with TopFD deconvolution. n=3 technical replicates were examined over the four independent single LC-MS/MS runs (FI90s, ST90s, FI30s, and ST30s datasets) from the *E. coli* lysate sample. The (+) markers show the numbers from the replicates, and the bars show their average values. **(a)** the numbers of proteoform IDs (left) and proteins (right). FI and ST datasets were analyzed in the same way as Fig. 2a, except that the mass deconvolution (both MS1 and MS2) was done by TopFD instead of FLASHDeconv. **(b)** analog of **(a)** with precursor SNR filtration (SNR threshold = 1.0). The precursor SNRs were estimated using TopFD deconvoluted precursor masses. From the proteoform IDs, the ones with low precursor SNRs were discarded (see Supplementary Fig. 16-20 for low SNR precursors). Note that an isolation window often contains multiple precursors of distinct precursor SNRs. Thus, even though FLASHIda selects an isolation window containing a precursor of high SNR, TopFD could select another precursor of low SNR from the same isolation window. Such precursors are filtered out for FIs datasets. **(c)** analog of **(b)** where only MS1 deconvolution was done by FLASHDeconv, or equivalently FLASHIda reported precursor masses were used. The boost from FLASHIda is not well observed when TopFD is used for MS1 deconvolution, regardless of the use of SNR filtering. But when FLASHIda reported precursor masses are used, the boost is again observed as shown in **(c)**. Source data are provided as a Source Data file.

Supplementary Figure 16.

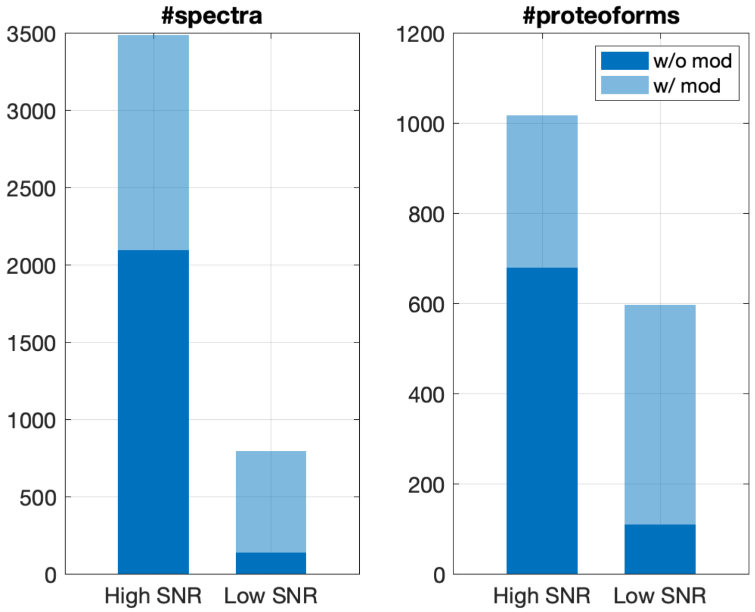

Supplementary Figure 16. Analog of Supplementary Fig. 5 for low precursor SNR identifications with TopFD deconvolution. We took the ST90 dataset analysis results from Supplementary Fig. 15 and divided them into two groups: ones of high-SNR precursors (precursor SNR>1; High SNR in x-axis) and the others of low-SNR precursors (precursor SNR<1; Low SNR). The left panel shows the number of identified spectra from each group and the right that of proteoform IDs. For the high SNR group, about 60% of spectral and 64% of proteoform IDs have no modification. But for the low SNR group, only 17% spectra and 18% proteoform IDs have no modification. Source data are provided as a Source Data file.

Supplementary Figure 17.

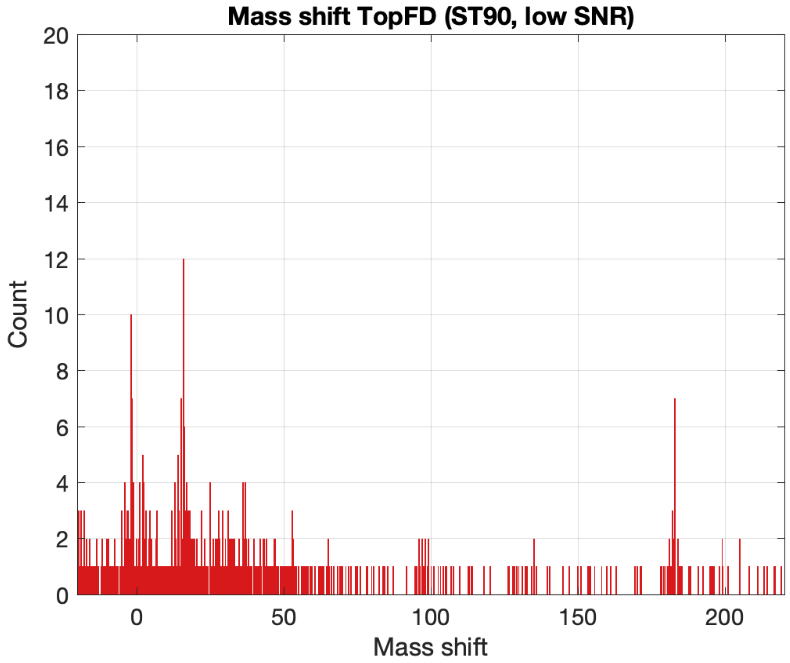

Supplementary Figure 17. Analog of Fig. 3e for low precursor SNR proteoform IDs with TopFD deconvolution. The histogram of the mass shifts from the low SNR group in Supplementary Fig. 16 is shown. In addition to the mass shifts corresponding to known modifications such as Oxidation and Intact disulfide bridge, a large number of non-specific mass shifts are observed as in Supplementary Fig. 8. Together with Supplementary Fig. 16, this shows that a large portion of low precursor SNR proteoform IDs are false positives. Source data are provided as a Source Data file.

Supplementary Figure 18.

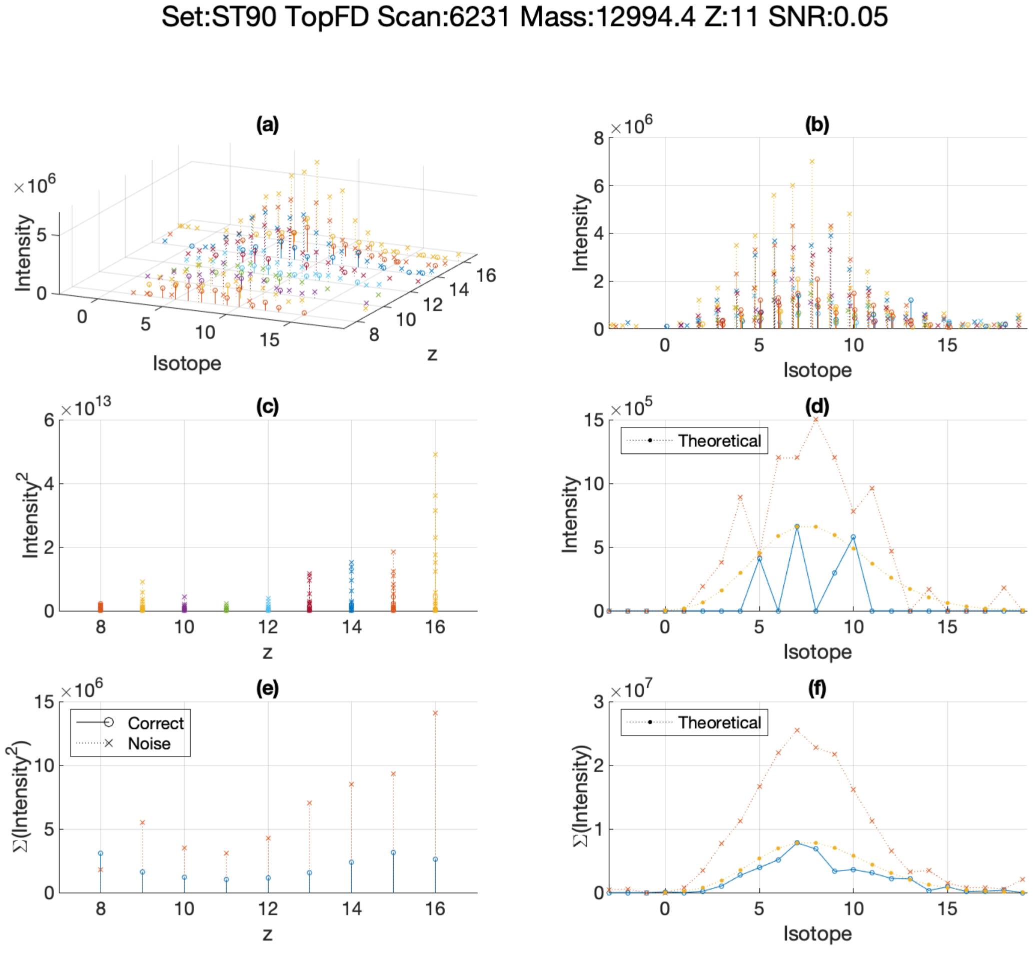

Supplementary Figure 18. Analog of Supplementary Fig. 2 (a-f) for ST90 dataset with TopFD deconvolution: an example of coelution. Signals of all low SNR precursors from ST90 dataset with TopFD deconvolution are deposited in MassIVE under accession number MSV000087484 [https://massive.ucsd.edu/ProteoSAFe/dataset.jsp?task=c2f199a1a5874350b48aea2fcb16c505] or under the digital object identifier [https://doi.org/10.25345/C5FJ9G].

Supplementary Figure 19.

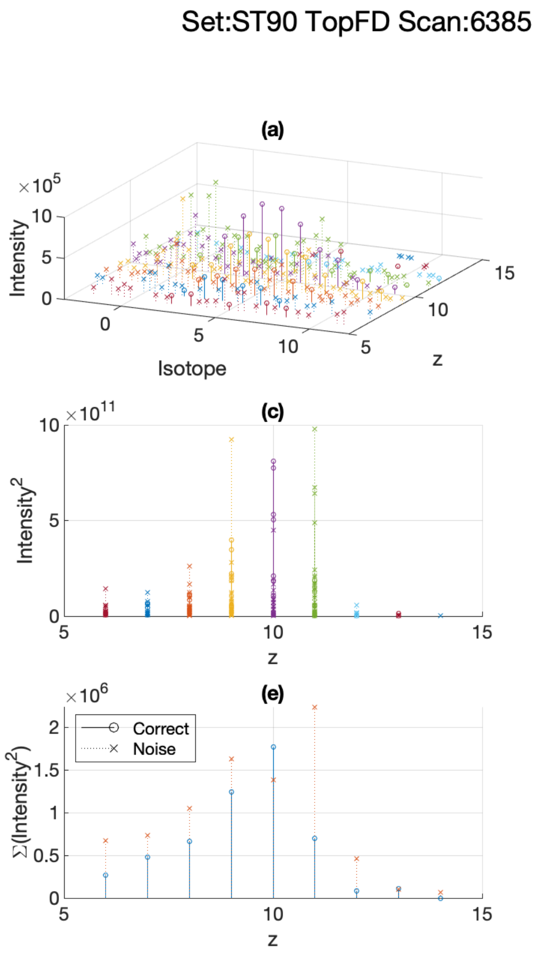

Supplementary Figure 19. Analog of Supplementary Fig. 2 (a-f) for ST90 dataset with TopFD deconvolution: another example of coelution.

Supplementary Figure 20.

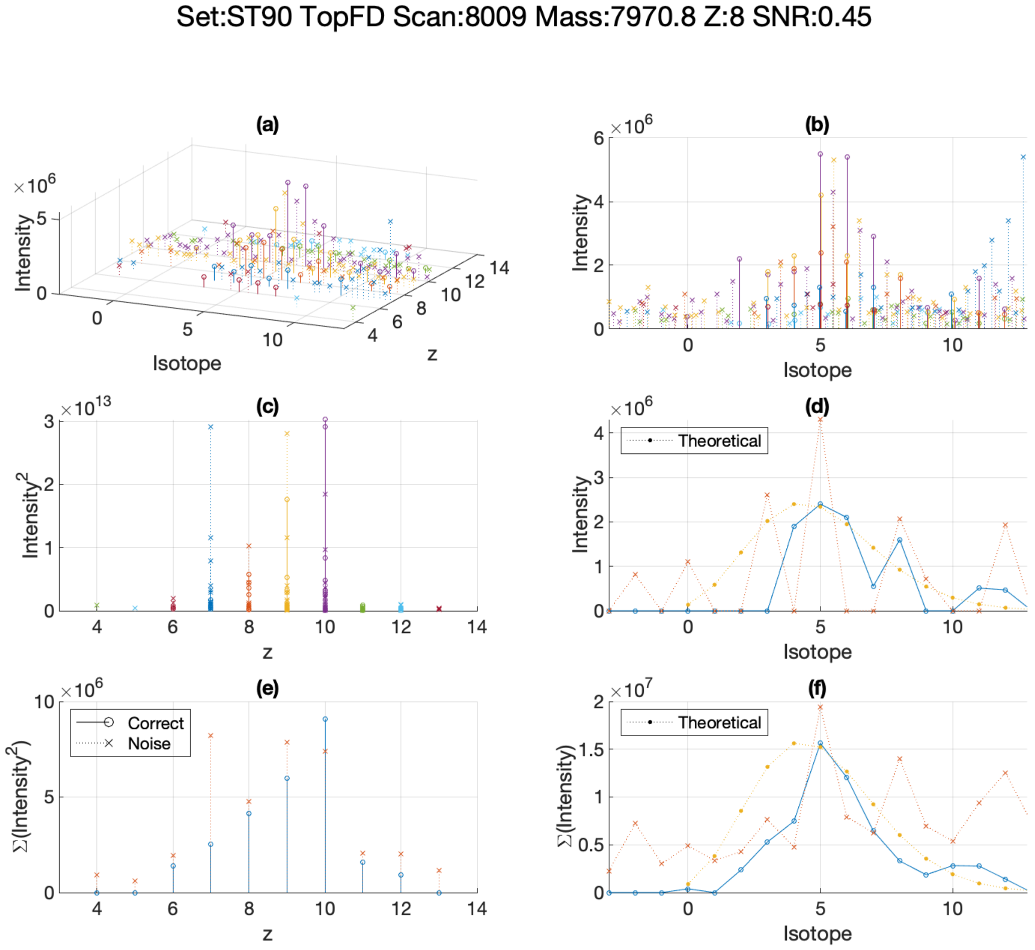

Supplementary Figure 20. Analog of Supplementary Fig. 2 (a-f) for ST90 dataset with TopFD deconvolution: an example of harmonic artifact.

Supplementary Figure 21.

(a)

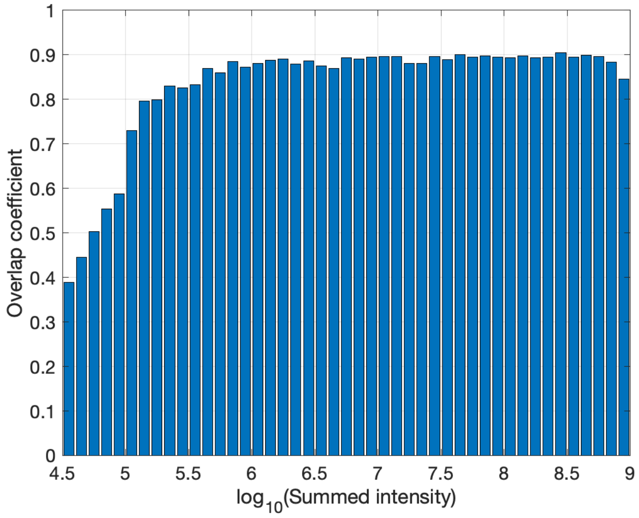

(b)

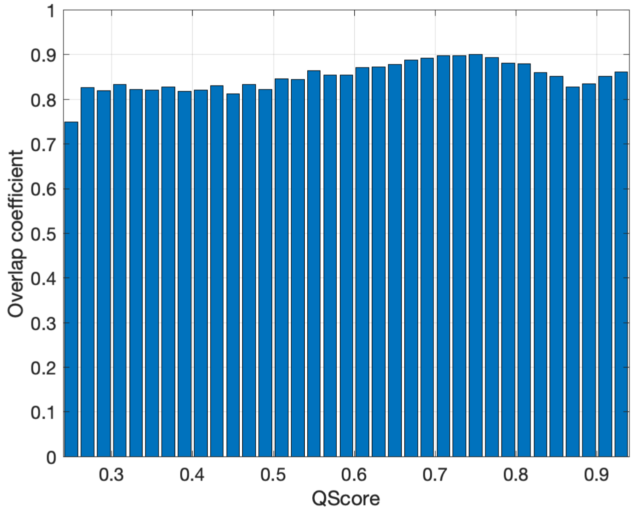

Supplementary Figure 21. Proteoform level overlap coefficients among FI90s along summed intensity (a) and QScore (b). Out of FI90s triplicates, possible three pairs were compared to each other (FI90 vs. FI90', FI90' vs. FI90", FI90" vs. FI90). Per each bin per pair, the overlap coefficient for the bin is calculated. Then the average overlap coefficient over three comparisons was drawn per bin. To calculate the overlap coefficient for a bin, the proteoform IDs corresponding to the bin from one set were collected. Then the collected ones were compared against all the proteoform IDs in the other set. In this way, the measurement is not affected by the QScore or intensity calculation consistency. But this calculation makes the overlap coefficients shown here (0.8-0.9) a bit higher than the overall overlap coefficient in Fig. 3a (0.63-0.65). Source data are provided as a Source Data file.

Supplementary Figure 22.

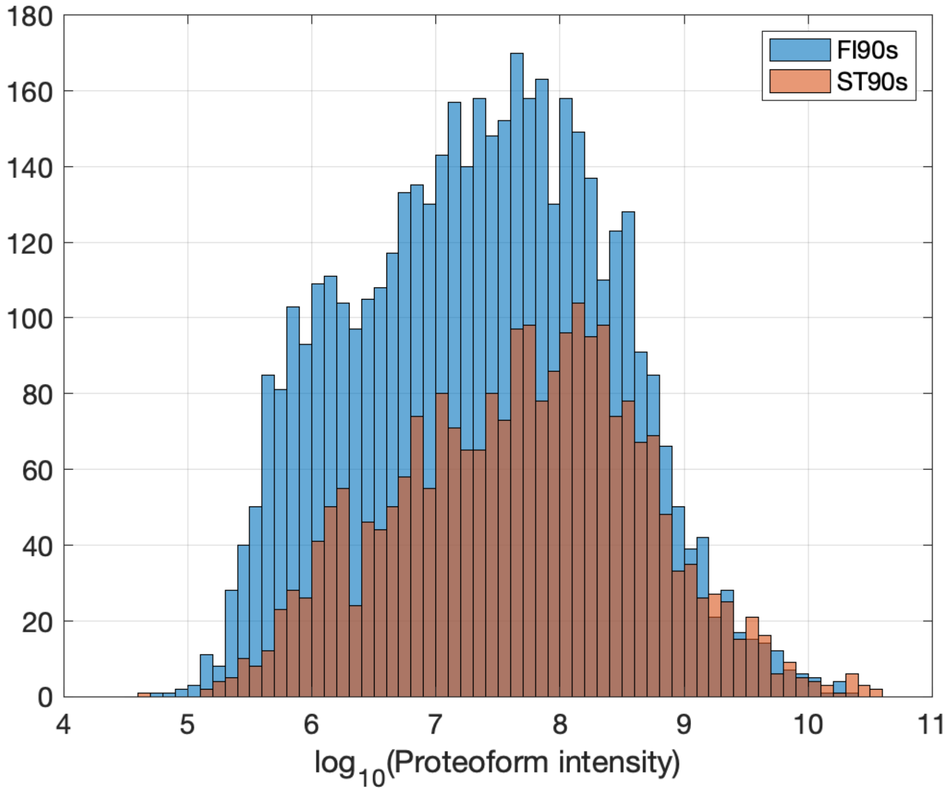

Supplementary Figure 22. The histogram of $\log_{10}$(proteoform intensity) for FT90s and ST90s datasets. The proteoform intensity is measured by the mass feature area of each proteoform ID reported by FLASHDeconv. It is clearly shown that the proteoform ID boost by FLASHIda is mainly obtained from low intensity proteoforms. Source data are provided as a Source Data file.

Supplementary Figure 23.

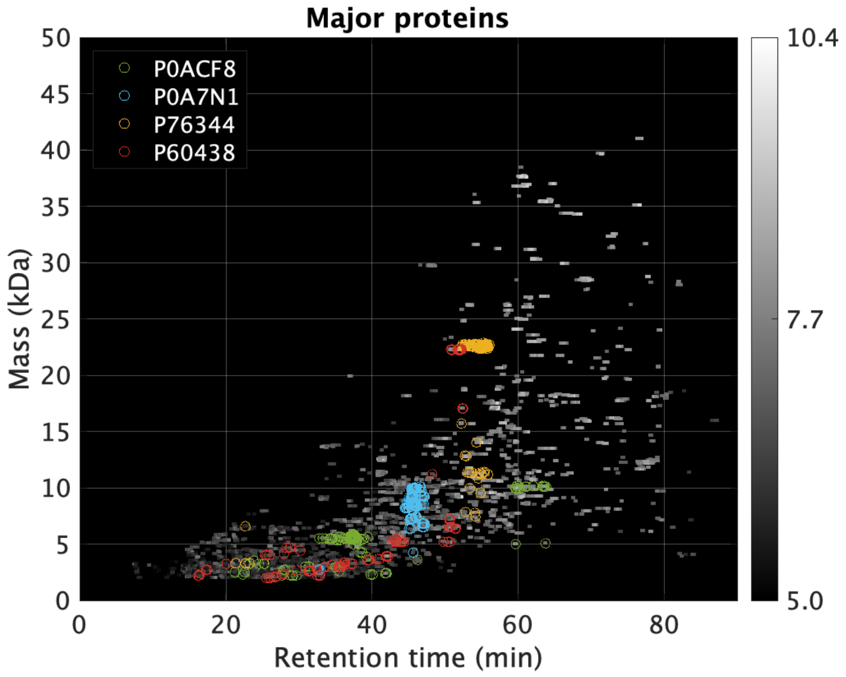

Supplementary Figure 23. Distribution of the proteoform IDs (identified in FI90s datasets) of the selected four proteins (UniProtKB: P0ACF8, P0A7N1, P76344, P60438) in RT-mass plane. Four proteins with highest proteoform ID heterogeneity have been selected from FI90s datasets: DNA-binding protein H-NS (UniProtKB: P0ACF8), 50S ribosomal protein L31 type B (UniProtKB: P0A7N1), metal-binding protein ZinT (UniProtKB: P76344), and 50S ribosomal protein L3 (UniProtKB: P60438). The proteoform IDs are color coded on the RT-mass plane along with other proteoform IDs in gray.

Supplementary Figure 24.

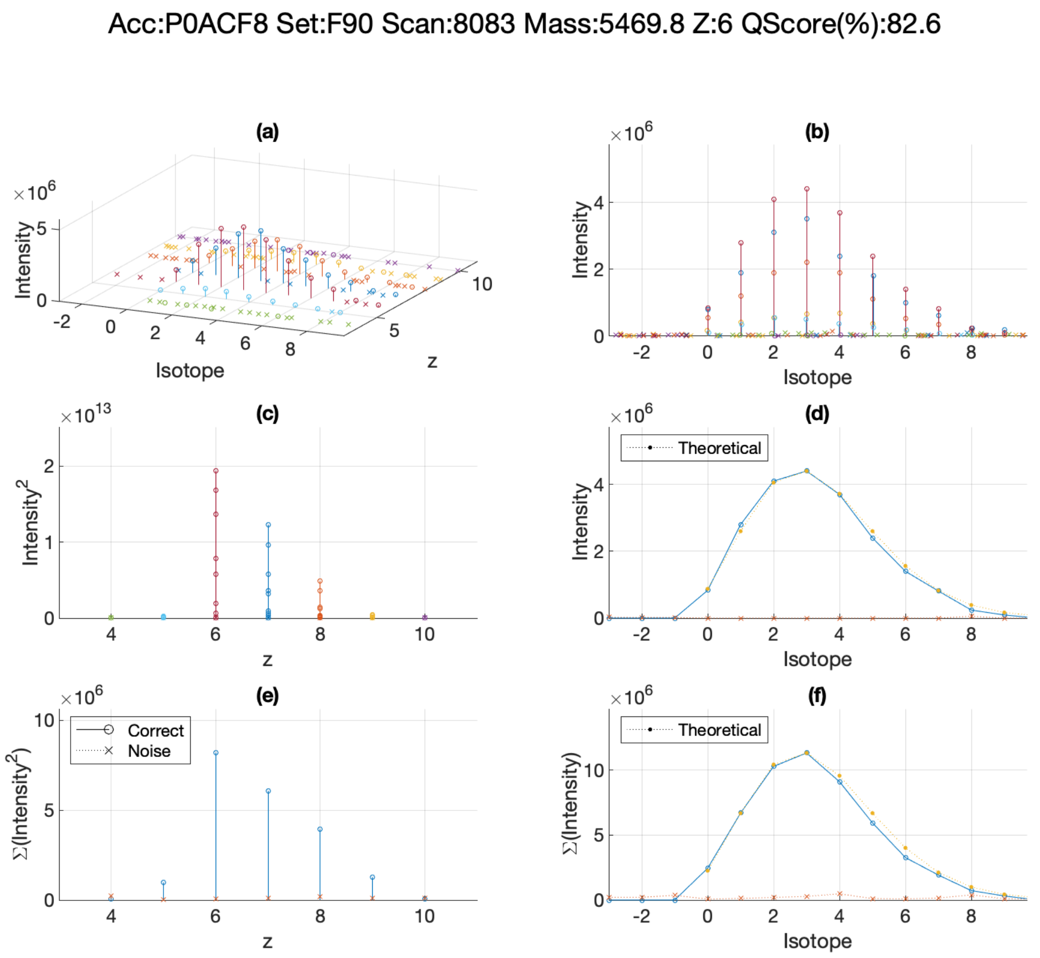

(g)

```
... 60 amino acid residues are skipped at the N-terminus ...

 61  Y R E M L I A D G I   D P N E L L N S L A   A V K S G T K A K R  90

 91  A Q R P A K Y S Y V   D E N G E T K T W T   G Q G R T P A V I K 120
             15.9976
121  K A M D E Q G K S L   D D F L I K Q                             137
```

Supplementary Figure 24. Analog of Supplementary Fig. 2 (a-f) and annotated sequence (g) for a DNA-binding protein H-NS (UniProtKB: P0ACF8) proteoform ID. MS1 signals of all proteoform IDs from the major four proteins are deposited in MassIVE under accession number MSV000087484 [https://massive.ucsd.edu/ProteoSAFe/dataset.jsp?task=c2f199a1a5874350b48aea2fcb16c505] or under the digital object identifier [https://doi.org/10.25345/C5FJ9G].

Supplementary Figure 25.

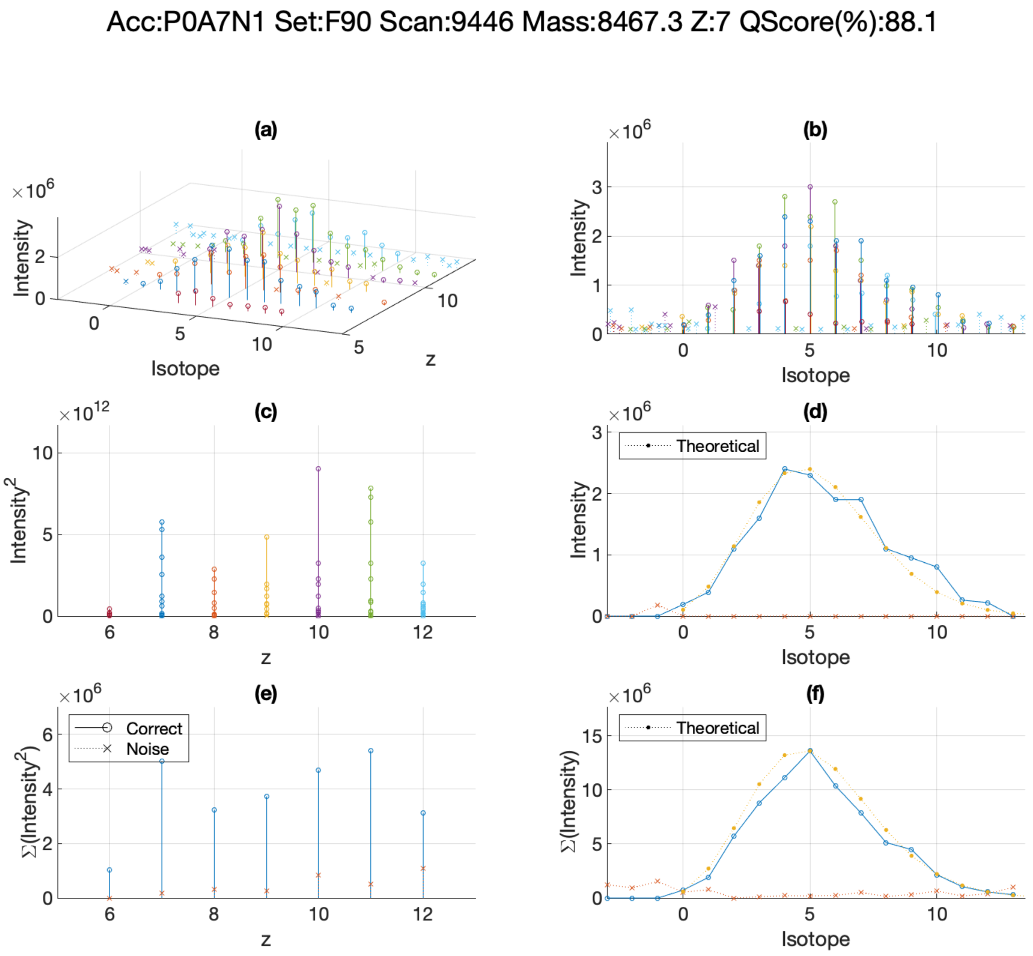

(g)

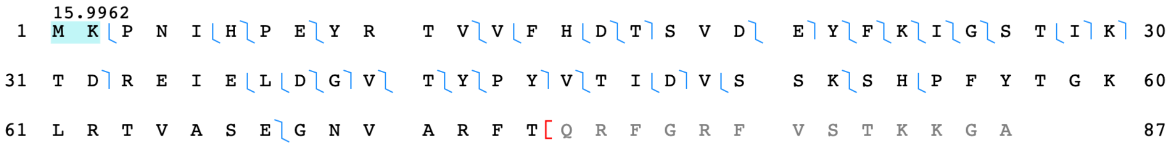

Supplementary Figure 25. Analog of Supplementary Fig. 2 (a-f) and annotated sequence (g) for a 50S ribosomal protein L31 type B (UniProtKB: P0A7N1) proteoform ID.

Supplementary Figure 26.

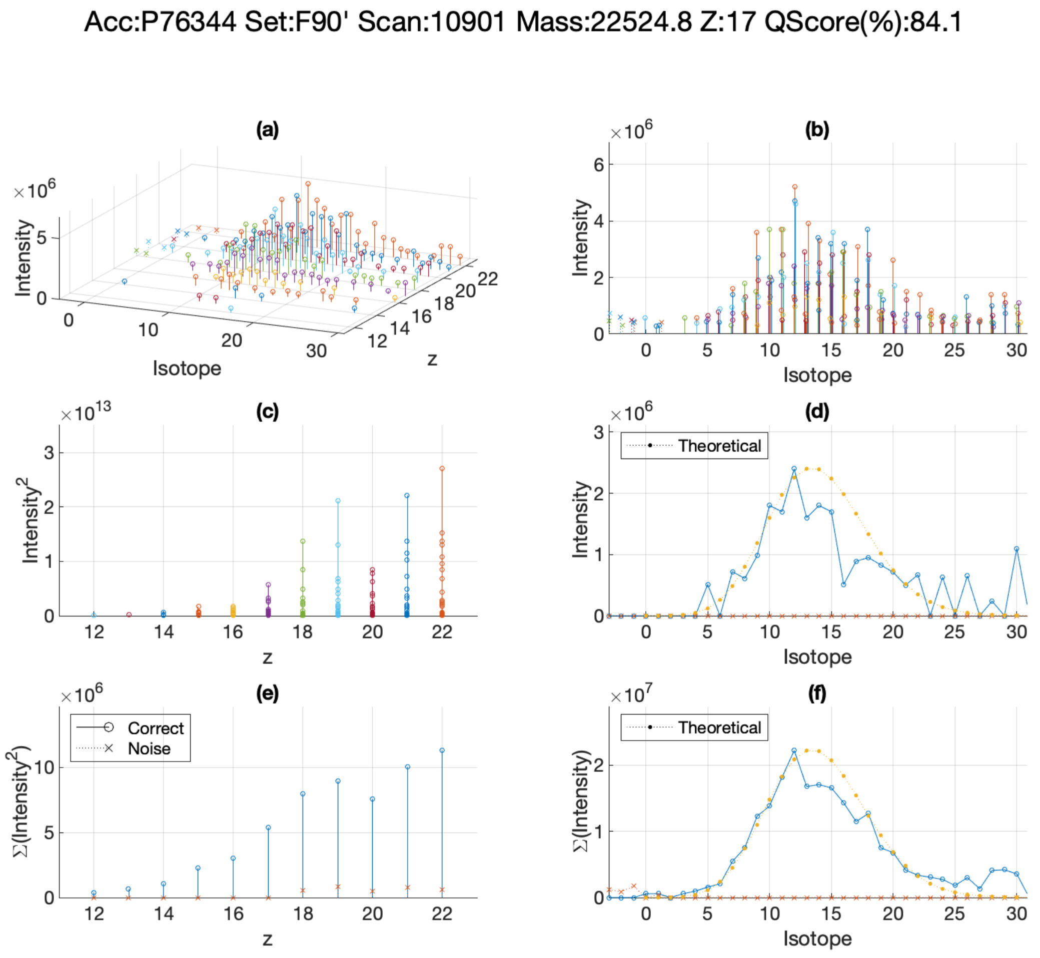

(g)

```
1    M A I R L Y K L A V   A L G V F I V S A P   A F S H G H H S H G   30
31   K P L T E V E Q K A   A N G V F D D A N V   Q N R T L S D W D G   60
                196.9935
61   V W Q S V Y P L L Q   S G K L D P V F Q K   K A D A D K T K T F   90
91   A E I K D Y Y H K G   Y A T D I E M I G I   E D G I V E F H R N   120
121  N E T T S C K Y D Y   D G Y K I L T Y K S   G K K G V R Y L F E   150
151  C K D P E S K A P K   Y I Q F S D H I I A   P R K S S H F H I F   180
181  M G N D S Q Q S L L   N E M E N W P T Y Y   P Y Q L S S E E V V   210
211  E E M M S H                                                       216
```

Supplementary Figure 26. Analog of Supplementary Fig. 2 (a-f) and annotated sequence (g) for a metal-binding protein ZinT (UniProtKB: P76344) proteoform ID.

Supplementary Figure 27.

Acc:P60438 Set:F90' Scan:10117 Mass:22259.8 Z:30 QScore(%):85.4

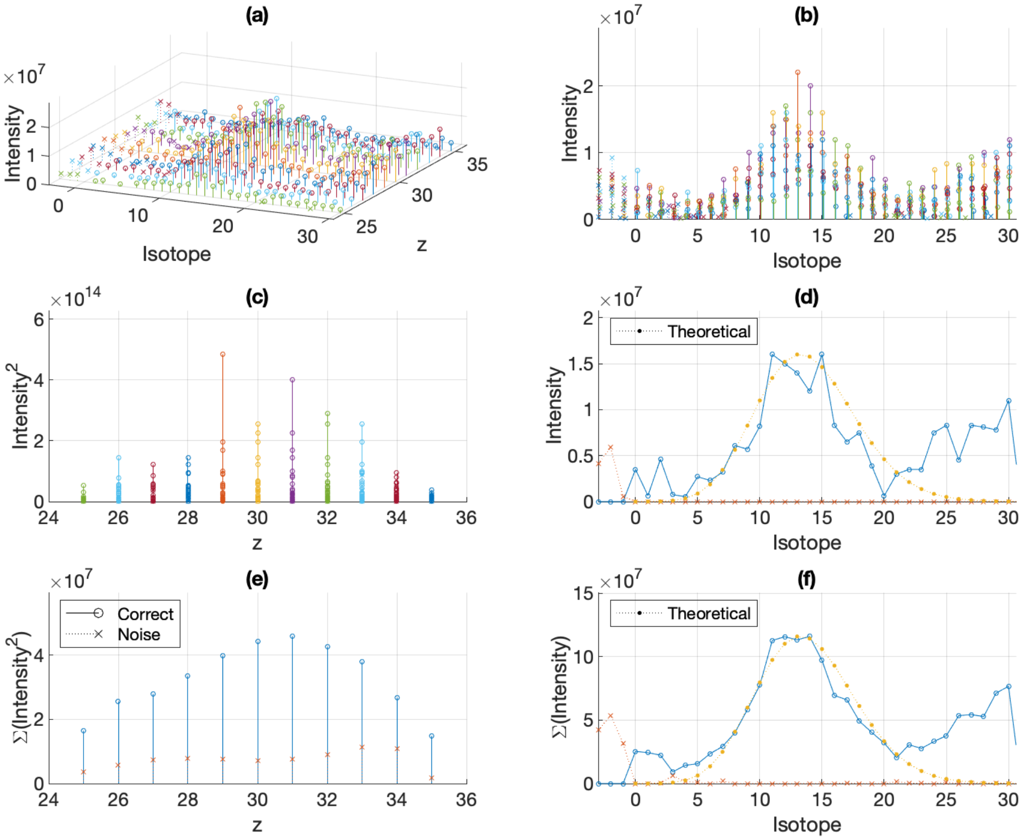

(g)

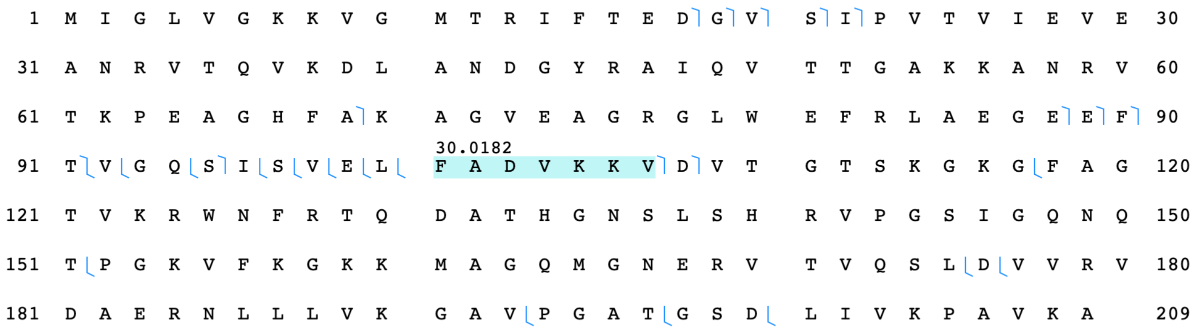

Supplementary Figure 27. Analog of Supplementary Fig. 2 (a-f) and annotated sequence (g) for a 50S ribosomal protein L3 (UniProtKB: P60438) proteoform ID.

Supplementary Table 1.

Name	Mass	Amino acid specificity	Position specificity	Unimod Acc
Acetylation	42.010565	K	any	1
Phosphorylation	79.966331	S,T,Y	any	21
Oxidation	15.994915	C,P,K,D,N,R,Y	any	35
Methylation	14.01565	C,K,R,H,D,E,N,Q	any	34
Di-oxidation	31.989829	M	any	425
Half of a disulfide bridge	-1.007825	C	any	374
Formylation	27.994915	M	N-term	122
AEBS	183.035399	H,K,S	any	276

Supplementary Table 1. Candidate modifications used in TopPIC search for analysis of FI90s and ST90s datasets to exclude chemical adducts and false positive modifications. Supplementary Data 14 is the TopPIC input file for these eight modifications. Supplementary Data 15 provides the TopPIC search results with these modifications.
