# Supplementary material for: FLASHIda enables intelligent data acquisition for top–down proteomics to boost proteoform identification counts
Source: Nat Commun. 2022 Jul 29;13:4407. doi: 10.1038/s41467-022-31922-z (PMC9338294; doi:10.1038/s41467-022-31922-z)
Supplement: Supplementary file 3 — Description of Additional Supplementary Files [file 41467_2022_31922_MOESM3_ESM.docx]

File Name: Supplementary Data 1.xlsx

Description: **Identification results for ST90 dataset with intentionally introduced precursor mass errors.**

File Name: Supplementary Data 2.fasta

Description: ***E. coli* proteome fasta file used for searches.**

File Name: Supplementary Data 3.xlsx

Description: **Proteoform IDs of FI and ST datasets.**

File Name: Supplementary Data 4.xlsx

Description: **PrSM IDs of FI and ST datasets.**

File Name: Supplementary Data 5.xlsx

Description: **Proteoform IDs of FI and ST datasets with TopFD deconvolution.**

File Name: Supplementary Data 6.xlsx

Description: **Proteoform IDs of FI and ST datasets with TopFD deconvolution and precursor SNR filtration.**

File Name: Supplementary Data 7.xlsx

Description: **Proteoform IDs of FI datasets with TopFD MS2 deconvolution (MS1 by FLASHDeconv).**

File Name: Supplementary Data 8.xlsx

Description: **PrSM identification results of FI and ST datasets with TopFD deconvolution.**

File Name: Supplementary Data 9.xlsx

Description: **PrSM identification results of FI and ST datasets with TopFD deconvolution and precursor SNR filtration.**

File Name: Supplementary Data 10.xlsx

Description: **PrSM identification results of FI datasets with TopFD MS2 deconvolution (MS1 by FLASHIda).**

File Name: Supplementary Data 11.xlsx

Description: **Proteins that are not identified in FI90s datasets out of previously reported 50 abundant *E. coli* proteins.**

File Name: Supplementary Data 12.xlsx

Description: **Gene ontology (GO) term analysis results for the identified proteins in FI90s and ST90s datasets.**

File Name: Supplementary Data 13.xlsx

Description: **Proteoform IDs (identified in FI datasets) of the selected four proteins (UniProtKB: P0ACF8, P0A7N1, P76344, P60438) and possible interpretations thereof.**

File Name: Supplementary Data 14.txt

Description: **TopPIC input modification file for the eight modifications (see Supplementary Table 1) used in TopPIC searches for Supplementary Data 15.**

File Name: Supplementary Data 15.xlsx

Description: **Proteoforms IDs (in FI90s and ST90s datasets) reported in TopPIC searches with eight candidate modifications in Supplementary Table 1.**

File Name: Supplementary Data 16.xlsx

Description: **Training dataset for QScore logistic regression.**
