# Supplementary material for: FLASHIda enables intelligent data acquisition for top–down proteomics to boost proteoform identification counts
Source: Nat Commun. 2022 Jul 29;13:4407. doi: 10.1038/s41467-022-31922-z (PMC9338294; doi:10.1038/s41467-022-31922-z)
Supplement: Supplementary file 20 — Reporting summary [file 41467_2022_31922_MOESM20_ESM.pdf]

nature portfolio

Corresponding author(s): Kyowon Jeong
Oliver Kohlbacher

Last updated by author(s): May 2, 2022

Reporting Summary

Nature Portfolio wishes to improve the reproducibility of the work that we publish. This form provides structure for consistency and transparency in reporting. For further information on Nature Portfolio policies, see our Editorial Policies and the Editorial Policy Checklist.

Statistics

For all statistical analyses, confirm that the following items are present in the figure legend, table legend, main text, or Methods section.

n/a	Confirmed	
☐	☒	The exact sample size (*n*) for each experimental group/condition, given as a discrete number and unit of measurement
☐	☒	A statement on whether measurements were taken from distinct samples or whether the same sample was measured repeatedly
☐	☒	The statistical test(s) used AND whether they are one- or two-sided<br>*Only common tests should be described solely by name; describe more complex techniques in the Methods section.*
☐	☒	A description of all covariates tested
☐	☒	A description of any assumptions or corrections, such as tests of normality and adjustment for multiple comparisons
☐	☒	A full description of the statistical parameters including central tendency (e.g. means) or other basic estimates (e.g. regression coefficient) AND variation (e.g. standard deviation) or associated estimates of uncertainty (e.g. confidence intervals)
☒	☐	For null hypothesis testing, the test statistic (e.g. *F*, *t*, *r*) with confidence intervals, effect sizes, degrees of freedom and *P* value noted<br>*Give P values as exact values whenever suitable.*
☒	☐	For Bayesian analysis, information on the choice of priors and Markov chain Monte Carlo settings
☐	☒	For hierarchical and complex designs, identification of the appropriate level for tests and full reporting of outcomes
☒	☐	Estimates of effect sizes (e.g. Cohen's *d*, Pearson's *r*), indicating how they were calculated

Our web collection on statistics for biologists contains articles on many of the points above.

Software and code

Policy information about availability of computer code

Data collection	FLASHIda (beta version): https://OpenMS.de/FLASHIda Github repository: https://github.com/caetera/FlashIda
Data analysis	ProteoWizard msconvert (version 3.0.20186-dd907d75)<br>FLASHDeconv (version 2.0 beta)<br>TopPIC (version 1.4.4)<br>Weka software (version 3.9.4)

For manuscripts utilizing custom algorithms or software that are central to the research but not yet described in published literature, software must be made available to editors and reviewers. We strongly encourage code deposition in a community repository (e.g. GitHub). See the Nature Portfolio guidelines for submitting code & software for further information.

Data

Policy information about availability of data

All manuscripts must include a data availability statement. This statement should provide the following information, where applicable:

- Accession codes, unique identifiers, or web links for publicly available datasets
- A description of any restrictions on data availability
- For clinical datasets or third party data, please ensure that the statement adheres to our policy

The raw and converted mzML files for FI and ST triplicate datasets have been uploaded to MassIVE under accession number MSV000087484 [https://massive.ucsd.edu/ProteoSAFe/dataset.jsp?task=c2f199a1a5874350b48aea2fcb16c505] or under the digital object identifier [https://doi.org/10.25345/C5FJ9G], where the identification results and MS1 signal screenshots (related to Supplementary Fig. 2-4, 18-20, and 24-27) are also found. The E. coli (strain: K12 MG1655 i)

database in fasta format used for all searches was downloaded from SwissProt (downloaded 28.05.2020; Supplementary Data 2). Source data are provided with this paper.

Field-specific reporting

Please select the one below that is the best fit for your research. If you are not sure, read the appropriate sections before making your selection.

- [x] Life sciences
- [ ] Behavioural & social sciences
- [ ] Ecological, evolutionary & environmental sciences

For a reference copy of the document with all sections, see nature.com/documents/nr-reporting-summary-flat.pdf

Life sciences study design

All studies must disclose on these points even when the disclosure is negative.

Sample size	To benchmark FLASHIda, we generated four sets of nano-RPLC MS/MS single-runs from E. coli lysate: two sets using FLASHIda acquisition, and two using standard acquisition for intact proteins (Intact Protein Mode) on a Thermo Scientific Orbitrap Eclipse. One set from each acquisition has a gradient time of 30 min and another 90 min. Each set was measured in technical triplicate, thus a total twelve measurements were obtained.<br>For each acquisition, we used two different gradient times (short and long) to demonstrate that FLASHIda could be used to increase throughput without sacrificing the identification sensitivity as compared with the standard acquisition. For each gradient time, we prepared three technical replicates (not biological replicates) as the goal of the analysis was to compare acquisition methods while minimizing the any other possible sources of bias.
Data exclusions	No data were excluded from analysis.
Replication	For triplicates in each set, we observed very similar numbers of proteoform identifications, as clearly seen in Figure 2a. In terms of identification reproducibility, the overlap coefficients were 0.5-0.7 in proteoform level and >0.7 in protein level. This rather low overlap has been deeply discussed in the manuscript with possible interpretations.
Randomization	We ran FLASHIda and standard runs in randomized order. The order was decided by python random function.
Blinding	Blinding is not possible as we need to know if each dataset was acquired by FLASHIda or by standard.

Reporting for specific materials, systems and methods

We require information from authors about some types of materials, experimental systems and methods used in many studies. Here, indicate whether each material, system or method listed is relevant to your study. If you are not sure if a list item applies to your research, read the appropriate section before selecting a response.

Materials & experimental systems

n/a	Involved in the study	
x		Antibodies
x		Eukaryotic cell lines
x		Palaeontology and archaeology
x		Animals and other organisms
x		Human research participants
x		Clinical data
x		Dual use research of concern

Methods

n/a	Involved in the study	
x		ChIP-seq
x		Flow cytometry
x		MRI-based neuroimaging
